# Assessing the relationship between the human learned helplessness depression model and anhedonia

**DOI:** 10.1371/journal.pone.0249056

**Published:** 2021-03-30

**Authors:** Xin Song, Iris Vilares

**Affiliations:** Department of Psychology, University of Minnesota, Minneapolis, Minnesota, United States of America; University of Pittsburgh, UNITED STATES

## Abstract

The learned helplessness (LH) model is one of the most commonly used acute stress models to explain depression and it has shown good face and predictive validity. However, despite being able to induce depressed-like behaviors and corresponding psychophysiological changes, there is little evidence showing that the LH paradigm can produce anhedonia, a core symptom seen in all forms of depression in humans. So far a couple of studies showed that rodents bred for helplessness develop anhedonic-like behaviors in response to stress; yet, to the best of our knowledge, no similar human research has tried to investigate the direct relationship between the LH model and anhedonia. In the present study, we use a modified version of the original LH task to experimentally and temporarily induce learned helplessness in college students and then examine if the human LH paradigm induces anhedonia. We aim to 1: address the ill-defined connection between the LH model and anhedonia, and 2: directly assess helplessness in humans as opposed to the majority of non-human animal subjects used in the helplessness literature. We believe that our study will fill an important gap in the learned helplessness model literature, and will advance our understanding of the relationship between depression and perceived control, as well as place limitations to what can and cannot be inferred from non-human animal data in this topic.

## Introduction

Major Depressive Disorder (MDD) is a leading cause of disability, with around 300 million people afflicted with depression worldwide, according to the World Health Organization [1]. Symptoms of depression include heightened negative affect (negative moods, e.g., feeling nervous, scared, and guilt, etc.) and diminished positive affect (positive moods, e.g., feeling less motivated, loss of pleasure, harder to concentrate, etc.). In addition to causing personal distress, depression also plays a large role in suicide attempts, with one-half of all suicide attempts linked to depression and nearly 20% lifetime suicide risk if left untreated [2]. Despite the large volume of studies devoted to depression research, our understanding of depression is still limited to this day, partially because depression is a heterogeneous disease that varies in symptomologies and underlying pathophysiology [3, 4]. Because the fundamental etiology of depression is not clear, recreating a perfect animal depression model that captures every and all aspects of the disease is nearly impossible. Nonetheless, psychologists proposed various animal models in the past decades in the hope to explain the formation of some core phenotypic characterizations that we see in human depression patients [5, 6]. Central to the animal depression models, some of the most widely used ones are the learned helplessness (LH) model, the chronic mild stress (CMS) model, the early life stress model, and the social defeat model [5]. These models simulate different processes that may lead to the development of depression, and each paradigm produces a set of behavioral features in animals that resemble a depressed-like state in humans. Although the animal models vary in the extent to which they resemble human depressive states, a disease model is only considered a good model when it has good face validity, construct validity, and predictive validity [7, 8]; in other words, a good animal depression model should have the following characteristics: (*1*) produce behavioral changes that are similar to those seen in human depression; (*2*) the changes can be objectively measured; (*3*) the changes can be reversed by treatments used to treat depression. An ideal depression model should recreate the complex, multidimensional symptom profiles of the human depression state. In this study, we aim to examine the learned helplessness depression model and anhedonia, a key symptom in depression.

### Development of the learned helpless model

Among the animal depression paradigms, the learned helplessness (LH) model is one of the most widely researched, with a large number of studies devoted to this paradigm over the past few decades. Originally proposed by Seligman and Maier in 1967, the LH paradigm demonstrates the phenomenon that after being exposed to unpredictable/inescapable aversive electric shocks, animals show impaired escape learning even in a new, controllable environment [9]. The original LH experiment on dogs and rodents is then followed up by a series of similar experimentations on humans and scientists quickly found parallels between non-human animals and humans: following unpredictable and uncontrollable aversive treatments (either loud tones or unsolvable cognitive problems), people demonstrated behavioral deficits similar to the ones seen in other animals [10, 11]. The above mention research explains that the theoretical mechanism of the observed behavioral deficits roots from maladaptive cognition: the perception of response-outcome independence generates a sense of uncontrollability, and the perceived uncontrollability prevents animals and humans from actively coping in a new stressful situation. This theory suggests that response-outcome non-contingency is the central construct to helplessness, and the formation of helplessness is not likely due to mere exposure to the stressor, as later studies showed people develop performance deficits following unsolvable anagrams problems but not controllable shocks [12]. People started to consider the LH as a laboratory model for clinical depression because lab-induced LH can produce many depression-like phenotypes such as reduced psychomotor responses, fatigue, etc. [13]. Besides behavioral changes, the LH paradigm has also shown to be able to alter psychophysiological functions in humans and non-human animals, such as changes in hypothalamic-pituitary-adrenal (HPA) axis activities [14] and sleep disturbance [15], both of which resemble human depression characteristics and thus contribute to the face validity of the LH model. The LH model also has good predictive validity because helpless animals have been found to respond to most kinds of antidepressants [16–18]. With overall good face and predictive value, the LH model has contributed to several lines of pathophysiological concepts in clinical research and has brought forward new prevention and treatment methods for various neurological disorders [19].

### Critiques of the LH theory and model comparison

In human LH experiments, uncontrollable aversive events are often found to produce behavioral impairments, but this finding is not universal, and the conditions in which humans develop helplessness is less certain in comparison to non-human animals. Performance impairments are seen in some tests but not others [12, 20]; also, helplessness is found to be rather context-and-task specific in some studies, as behavioral deficits fail to transfer across situations [20]. In light of this, Abramson et al. [12] reformulated the original LH theory and explained that besides uncontrollability, we also need to also reference to human cognition and attributional styles to account for all effects we see in human LH experiments. Abramson’s version states that participants learn to be helpless if they perceive the response-outcome non-contingency, but only if they make a correct attribution as the cause of their failure. More specifically, their study suggested that participants who had global attributional styles showed helplessness in both similar and new contexts; on the other hand, participants who had a specific attributional style only showed helplessness in a similar context, but this helplessness did not transfer to a new context. The new LH theory can account for most human results that could not be explained by the original LH theory, yet in doing so, it creates a divergence between non-human animal and human research because the reformulated model is considerably less testable in non-human animals [21, 22].

As a depression model, the standard LH procedure can reliably produce lowered voluntary response initiation, a symptom that is pervasive in some forms of depressions but not others [21]. The LH model is sometimes compared with the chronic mild stress (CMS) model, originally devised by Katz [23] and later modified by Willner [24, 25], as they both have high face validity and predictive validity [5, 26]. In the CMS model, animals that are exposed to chronic unpredictable micro-stressors over a time course from 3 weeks to 3 months later show decreased responses to rewards, measured by reduced sucrose preference. The reduced sensitivity to pleasurable sucrose solution resembles the loss of interest observed in the human depression and hence considered an animal behavioral correlate of anhedonia, namely, the inability to experience pleasurable situations. While psychomotor unresponsiveness is a defining feature in the LH model, anhedonia is not; on the other hand, the CMS model has shown its ability to alter hedonic capacity and produce anhedonia, the hallmark of depression that distinguishes depression from anxiety disorders. Extensive experimentations have established a strong connection between chronic mild stress and the development of anhedonia [24, 25]; however, the connection between the LH model and anhedonia remains unclear.

When comparing these two models, the following questions arise: do the short stress sessions used in the LH paradigm really resemble the circumstances that are necessary for the formation of depression, and can the LH model cause persistent behavioral and mood alterations, anhedonia included, days or even months after the sessions are concluded? Previous studies investigating the time course of the LH paradigm have shown that the failure to escape persisted only for a short time after the animals are exposed to uncontrollable shocks; specifically, the depression of active escape responses disappeared after 48 h to 72 h [9, 27]. Also, the neurochemical changes induced by uncontrollable stressors persisted only for a few days [28], showing a similar time course to the fast-disappearing behavioral deficiency. This evidence shows that experimentally induced LH only has transient effects on animal/human behaviors and does not always resemble the sustained changes seen in human depression [9, 27, 28]. Some argue that the unpredictable and uncontrollable stress schedule used in the LH experiment is uniquely linked to the formation of depression, but also a number of other anxiety disorders including post-traumatic stress disorder (PTSD), and that the LH model might be a better model for PTSD [29, 30]. The rationale behind modeling LH for PTSD is based on the similarity of events (unpredictable and uncontrollable stimuli) that lead to the development of learned helplessness and PTSD, and the similarities in physiologic and behavioral alterations in the two [30]. Using the LH theory to explain both depression and PTSD is not unreasonable because there are high comorbidity rate and overlapping symptoms between the two disorders [31, 32], and the learned helplessness model has been shown to produce some behavioral sequelae that are shared between depression and PTSD [33, 34]. However, despite the substantial comorbidity rate and many shared symptoms, depression and PTSD have different determinants [35], symptomatologies [34], as well as distinguished diagnosis criteria [31], and therefore should not be confused with one another. Even though some studies also reported anhedonia and emotional numbing in PTSD patients [36, 37], it is possible that the observed diminished positive affect is not a result of PTSD itself but instead a manifestation of the concomitant depression. Due to the substantial overlapping in symptoms between depression and PTSD, it is unclear to what extent the LH model recreates aspects that are purportedly unique to depression and not PTSD.

Over the years, the LH model has continued to show great translational value and contributes much to our understanding of depression and the development of new antidepressants [38]. But just like all models, the LH model has its limitations: it can reliably produce psychomotor unresponsiveness and heightened stress and fear responses, but not some other core symptoms of depression like anhedonia, so it is sometimes considered a generic stress model that is not purportedly unique to depression. And because the gradual development of anhedonia is a common and essential attribute to all forms of depression, the weak link between the LH model and anhedonia weakens the face validity of the LH model. While there is an enormous body of evidence in the existing literature supporting the relationship between the CMS model and anhedonia [24–26], the connection between the LH model and hedonic capacity remains a topic still under-researched. The LH paradigm seems to show reliable behavioral and cognitive deficits in humans, but the emotion deficits (anhedonia) are less discussed. Even though the LH paradigm produces a wide range of psychophysiological and phenotypic changes in humans and other animals [14, 15, 17, 18, 26], it is unclear whether the LH procedure alone is sufficient to induce anhedonia in humans and non-human animals alike.

Recent studies have also shown that rodents that are genetically predisposed to helplessness develop an anhedonic phenotype and other depression symptomologies in response to acute stress [26, 39–41]. But in general, while studies of the LH model and depression continue to advance in animal research, it is less discussed in human research in the past decades. Earlier studies in the 1970s and 1980s tried to reproduce the animal findings in humans and the behavioral changes are generally well-replicated, such that human participants who experienced unpredictable aversive stimuli showed comparable impairments in escape learning and problem-solving even in new contexts [10, 11, 42–44]. Later reformulation of the LH theory by Abramson et al. [12] enriches the original theory and is able to better explain most of the LH experiments in humans. One study shows that acute stress can reduce reward responsiveness and produce anhedonic-like states in humans [45]; a more recent computational model using Bayesian learning has shown that LH procedure can cause reduced sensitivity to both aversive and appetitive stimuli alike [46] and that the LH paradigm has the potential to alter reward learning processes and possibly impact hedonic capacity in humans. There is also evidence that people who possess more LH traits show greater depressive symptoms, but no causal effect can be drawn and anhedonia is also not the main focus [47]. To the best of our knowledge, human studies that examine the direct connection between learned helplessness and anhedonia remain scarce in the existing literature. None of the human studies we know of has tried to experimentally induce anhedonia in humans using the full LH paradigm and the LH paradigm alone.

### The present study

In the present study, we want to further examine the validity of the LH model and investigate if the LH paradigm can induce anhedonia, the hallmark of depression; specifically, we will tackle the problem of whether the classic LH paradigm alone can produce an anhedonic state in humans. We do not expect the human LH task to cause a permanent change in a personality *trait*; rather, we are interested in seeing if experiencing uncontrollable, unpredictable stress will produce a change in the hedonic *state* in human participants. We expect to also see the LH paradigm produce an elevated anxiety state, in accordance with previous findings [29, 30]. To experimentally induce learned helplessness, we devise a computer LH task similar to the original human LH task [48].

In recent years, reward processing is shown to be not a unitary but a complex construct that can be broken down into several domains including motivation, learning, core hedonic response, etc [49]. Clinical assessments often times use anhedonia as an inclusive term and emphasize self-reported hedonic deficits vs other reward processing aspects, but recent research has pushed for more refined descriptions of anhedonia, as a recent study has evidence showing that anhedonia is more than just inability to experience pleasure [50]. Motivation and consummatory hedonic responses of anhedonia are oftentimes treated as equivalent yet research has shown that these two are distinct concepts. Also, studies have shown that different phases (anticipation and outcome phases) of reward processing involve different neural circuitries [51, 52], thus reward processing should be treated as a multi-dimensional construct that spans across different domains. For our study, we are primarily interested in the explicit hedonic experience and the reward sensitivity aspect of anhedonia. Anticipation and outcome are important future questions but are not the scope of this current study.

To assess hedonic capacity, we use a battery of pleasure scales as well as a behavioral probabilistic reward task/signal detection task (SDT) [53] to provide an independent behavioral measure to assess hedonic responses. The SDT is designed to measure response bias towards a rewarding stimulus, with a blunted response towards a reinforcer serving as an indicator of hedonic hypofunctioning.

### Hypotheses

We hypothesize that: (*1*) participants who experience uncontrollable, unpredictable stressors in the LH task will acquire learned helplessness and have impaired escape learning later in a new context; (*2*) participants who acquire learned helplessness will later show an anhedonic-like state, measured by lower scores in the *state* anhedonia surveys; participants who acquire learned helplessness will show not only *(3)* an elevated stress state, measured by higher scores in the *state* anxiety survey, but also *(4)* an anhedonic-like state, measured by a reduced positive bias in the probabilistic reward task; *(5)* the *state* anhedonia surveys and the probabilistic reward task will correlate well, and together, they should support our theory that the individuals who acquire learned helplessness will show an anhedonic state. As a secondary analysis, we will also analyze the *trait* anhedonia and anxiety scores, and we expect that individuals with higher *trait* anhedonia and anxiety scores will acquire learned helplessness faster and more readily, compared to those who have lower scores. Detailed research questions, hypotheses, and predicted outcomes, as well as the analysis pipeline are discussed in Table 1.

**Table 1:** Summary table of our research questions, hypotheses, analysis plans, and predicted outcomes.

Hypotheses	Statistical Analysis Plan	Predicted Outcome
**Primary research question: Can learned-helplessness induce an anhedonic-state in human participants?**		
1. The human learned-helplessness paradigm will induce learned-helplessness in human participants, evidenced by impaired escape learning in a new context.	To compare the average response latency of the 3 treatment groups across 6 blocks, a repeated measure, between factors ANOVA will be performed, with blocks as a within-subjects measure and treatment groups as a between-subjects measure. Post-hoc Scheffe tests will be performed in case of significant findings.	We predict that the result will align with our hypothesis that the participants from the yoked group, but not the master and control group, will show impaired escape learning in a new context.
2. The human learned-helplessness paradigm will induce an increased anhedonic state in human participants, evidenced by decreased hedonic scores in the *state* anhedonia surveys.	To compare the average scores on the anhedonia surveys for the 3 treatment groups, a one-way, repeated measure ANOVA will be performed, with participants as within-subject factors and treatment groups as between-subject factors. Post-hoc Tukey tests will be performed in case of significant findings.	We predict that the result will align with our hypothesis, such that the participants from the yoked group, but not the master and control group, will have decreased hedonic scores in the *state* anhedonia surveys.
3. The human learned-helplessness paradigm will induce an elevated anxiety state in human participants, evidenced by increased anxiety scores in the *state* anxiety survey.	To compare the average scores on the anhedonia surveys for the 3 treatment groups, a one-way, repeated measure ANOVA will be performed, with participants as within-subject factors and treatment groups as between-subject factors. Post-hoc Tukey tests will be performed in case of significant findings.	We predict that the result will align with our hypothesis, such that the participants from the yoked group, but not the master and control group, will have increased anxiety scores in the state anxiety survey
4. The human LH paradigm will induce an anhedonic state in human participants, measured by reduced positive bias in the signal detection task.	To compare the mean response bias for the 3 treatment groups across 4 blocks, a repeated measure, between-factors ANOVA will be performed, with blocks as within-subjects measure and treatment groups as the between-subjects measure. Post hoc Newman-Keuls tests will be performed in case of significant findings. 2. We will also conduct a Pearson Correlation test (or Spearman if assumptions are not met) to directly assess the relationship between the response latency from the LH task and response bias, combining the data from all 3 treatment groups.	We predict that the results will align with our hypothesis, such that the participants from the yoked group, but not the master and control group, will show reduced positive bias in the signal detection task. In addition, we expect a negative correlation between response latency and response bias, such that participants who have longer response latency will show more blunted response bias.
**Secondary research question 1: Will the self-reports and the behavioral measures of anhedonia correlate well with each other? And will the anhedonia surveys correlate well with one another?**		
1. The results we get from the signal detection task will be positively correlated with *state* anhedonia surveys; people who show reduced response bias in the signal detection task will have lower hedonic scores in all of the *state* anhedonia surveys.	To examine if the signal detection task correlates well with the anhedonia surveys, a Spearman correlation test will be performed.	We predict that there will be a positive correlation between the response bias and the scores in the anhedonia surveys, such that participants who show reduced response bias in the signal detection task will have lower hedonic scores in all of the *state* anhedonia surveys.
2. The results we get from the *state* anhedonia surveys will be positively correlated with one another.	To examine if the anhedonia surveys correlate well with one another, a Spearman correlation test will be used.	We predict that there will be a positive correlation between the 2 *state* anhedonia surveys; participants who report lower hedonic scores on one survey will also report lower hedonic scores on the other survey.
**Secondary research question 2: Will individuals’ hedonic *trait* and anxiety *trait* scores affect how readily they acquire learned-helplessness?**		
1. Individuals who have higher scores on the *trait* anhedonia surveys (showing less hedonic capacity), will acquire learned helplessness faster compared to those who have lower *trait* anhedonia scores.	A Spearman correlation test will be performed to assess the correlation between the hedonic traits of participants and how fast they acquire learned helplessness.	We predict a positive correlation between anhedonic *trait* scores and the speed of acquisition of learned helplessness.
2. Individuals who have higher *trait* anxiety scores on the anxiety surveys, indicating higher anxiety, will acquire learned-helplessness faster compared to those who have lower *trait* anxiety scores.	A Spearman correlation test will be performed to assess the correlation between the anxiety traits of participants and how fast they acquire learned helplessness.	We predict a positive correlation between anxiety *trait* scores and the speed of acquisition of learned helplessness.
**Secondary research question 3: How does attributional style contribute to the formation and transferability of learned helplessness?**		
Participants who have global attributional styles will show helplessness in both similar and new contexts; participants with a specific attributional style will only show helplessness in a similar context, but this helplessness may not transfer to a new context.	3 Spearman correlation tests will be performed to assess the correlation between attributional styles and the average response latency in the LH task, the correlation between attributional styles and average anhedonic scores across different anhedonia surveys, and the correlation between attributional styles and average response bias in the SDT task.	If no main effects are found in the LH or the SDT task in any of the treatment groups, we think attributional styles may account for the lack of effects in any one or more of our measures. We predict participants who do not show helplessness would have a specific attributional style (vs a global attributional style).
**Exploratory Analysis: What other strategies would the yoked group explore given their lack of control?**		
We anticipate that the yoked participants will likely explore different strategies given their lack of control. This may manifest in more keypresses during any given trials, more escape sequences attempted, and a decline in key presses after a certain number of trials, etc. We will perform exploratory analyses on the above-mentioned measure to assess participants’ exploratory behaviors.		

## Materials and methods

### Participants

All participants will be recruited through the online course extra credit system at our university. To qualify for the study, participants must be 18 years of age or older, have normal or corrected to normal vision and hearing, and report no past or present neurologic illness. Participants will complete five short surveys (details of surveys can be found in the following section) as well as two computer tasks in this experiment. Participants receive extra course credit upon completion of the experiment and will also have a chance to win a 100-dollar Amazon gift card.

### Sampling plan for primary research questions

#### Sampling plan for the LH task

Participants will be randomly assigned to 1 of the 3 treatment groups (details can be found in the following section) for the LH tasks. Based on the previous human learned helplessness results reported by Hiroto [48] and Thornton and Jacobs [54], the estimated effect size falls between 0.5 and 0.8, and it requires approximately 66 participants to reach an a priori power of 0.95. However, we believe that the actual effect size might be lower due to publication bias, and to maintain a priori power of 0.95 we believe we will need more participants so we use a smaller effect size in our power analysis (half of the one reported in the literature) to make sure that we have enough participants. An a priori power analysis was conducted using G*Power3 [55] to test the differences of 3 independent group means, tested across 6 trial blocks, using a repeated measure, between factors ANOVA test. With a medium effect size (*d* = 0.25) and an alpha of 0.05, and assuming a correlation among repeated samples of 0.5, we will need n = 150 to achieve a power of 0.95 or higher.

#### Sampling plan for the state-anhedonia and state-anxiety surveys

An a priori power analysis was conducted using G*Power3 to test the differences of 3 independent group means, tested twice for each group (pretest and posttest), using a repeated measure, between factors ANOVA test. With an effect size (*d* = 0.25) and an alpha of 0.05, and assuming a correlation among repeated samples of 0.5, we will need n = 189 to achieve a power of 0.95 or higher.

#### Sampling plan for the signal detection task

We are interested to see if the 3 treatment groups will perform differently in the signal detection task across 4 blocks. To obtain a medium effect size of 0.25, with an alpha of 0.05, for a repeated measure, between factors ANOVA test, we need n = 168 or higher to achieve a power of 0.95 or higher.

#### Sampling plan for the correlation between the LH task results and the SDT task results

In addition to directly comparing the means of the response bias among the 3 treatment groups, we hope to extract more information by combining the data from all the 3 treatment groups and study the correlation between the response latency of the LH task and the response bias of the signal detection task. We performed another power analysis using G*power3 to calculate the appropriate sample size needed for a correlation test. To achieve a medium to a large effect size of 0.25 and using an alpha of 0.05 for a bivariate normal model correlation test, we will need n = 168 (for a one-tailed correlation test, given that our hypotheses are directional).

#### Sampling plan summary

To account for the sample sizes we need for the above 4 measures, we choose the biggest estimated sample size (n = 189) as our minimal sample size. We choose to increase the total n to 200 in case we have drop-outs or missing data. We chose a medium to a large effect size of 0.25 for the correlations because we will also do individual differences analysis (as a secondary analysis) and that is very close to the suggested typical medium effect size of 0.2 [56]; but an effect size of 0.25 fits much better with our overall power analysis outcome as well as our data collection plan.

#### Data exclusion

Data from participants who fail to complete the entire experiment will not be included in the final data analysis. For the signal detection task: trials with reaction times (RT) longer than 2500 msec or shorter than 150 msec will be treated as inattentive trials and excluded. For each participant, trials with RT outside the range of mean ± 3 SD are excluded as outliers.

### Self-report surveys

#### Demographics questionnaire

This survey collects participants’ basic demographic information including such as age, gender, and ethnicity, etc.

#### Anhedonia questionnaires

Participants will fill out 1 survey to assess participants’ *trait* anhedonia (general state of anhedonia, “over the past few weeks” or “in general”) and 2 pleasure scales to assess their *state* anhedonia (current mood state/temporary anhedonia, “right now”). The three scales used in the experiment are the *Beck Depression Inventory-II* (BDI-II) [57], which includes an anhedonia subscale BDI-Anhedonic, the *Fawcett-Clark Pleasure Capacity Scale* (FCPS) [58], and the *Dimensional Anhedonia Rating Scale* (DARS) [59]. Due to the nature of the questionnaires, the BDI-Anhedonic is only administered once at the beginning of the experiment session, whereas the FCPS and the DARS scales are administered twice, once prior to and once after the learned helplessness task. We will use the BDI-Anhedonic as a general hedonic *trait* measurement, and the FCPS + DARS surveys as a hedonic *state* measurement before and after the learned helplessness task, to see if there is a change in the hedonic tone caused by the LH task.

The BDI-II is a 21-item questionnaire that is most widely used to assess depression severity. The BDI-Anhedonic subscale is created by summing all the responses from the 3 items that measure anhedonic symptoms (item # 4, loss of pleasure; item #12, loss of interest; item # 21, loss of interest in sex; see [53] for details). The BDI-Anhedonic is constructed to measure *trait* anhedonia (or extended anhedonic state, which lasts at least a few days) with questions asking participants to rate their pleasure capacity “in the past two weeks”. Higher scores on this scale suggest more severe anhedonic responses, meaning lower hedonic capacity.

The FCPS is a 36-item pleasure scale that assesses participants’ hedonic responses to hypothetical pleasurable situations based on their current mood state (e.g., “You sit watching a beautiful sunset in an isolated, untouched part of the world”), with higher scores indicating greater hedonic capacity. The FCPS assesses *state* anhedonia by asking participants to give their responses based on their feelings “at the current state”.

The DARS is a 17-item scale that measures the hedonic capacity across desire, motivation, effort, and consummatory pleasure domains (e.g., “Please list at least 2 of your favorite pastimes/hobbies that are Not primarily social”, “Please list at least2 of your favorite foods/drinks”); participants are asked to rank how much they would enjoy the items they list right now, with a higher score indicating greater hedonic capacity.

#### Anxiety questionnaire

Participants will fill out 1 questionnaire to report both their *trait* anxiety and *state* anxiety. The *State-Trait Anxiety Inventory* (STAI) is a 4-point Likert scale survey that consists of 40 items and two separate forms that measure both *state* anxiety (S-anxiety, anxiety about an event) and *trait* anxiety (T-anxiety, anxiety level as a personal characteristic), with 20 items for each type of anxiety [60]. Higher scores on the STAI indicate greater anxiety. The STAI is administered once at the beginning of the experiment session. After the learned helplessness task, we again collect the participants’ responses to only the S-anxiety portion in the STAI survey in order to see if there are any changes in the S-anxiety scores caused by the learned helplessness task.

#### Attributional style questionnaire

Participants will fill out one survey to report their individual attributional style at the beginning of the session. The Attributional Style Questionnaire (ASQ) is a self-report instrument that measures attributional styles for bad events and good events [61]. According to the reformulated learned helplessness model, depressive symptoms are associated with an attributional style in which uncontrollable bad events are attributed to internal (versus external), stable (versus unstable), and global (versus specific) causes. In the ASQ scale, participants are presented with 12 hypothetical scenarios (half good, half bad) and are asked to decide what the major cause of the situation would be if it happened to them. Participants then rate the cause along a 7-point Likert scale for each of the three causal dimensions.

### The Learned-Helplessness (LH) task

To experimentally induce learned helplessness in human participants, we adapted the original human version of the learned helplessness (LH) task [48, 62] using a triadic design and a computerized modification of the original task. The triadic design, used in the original and almost all of the following learned helplessness study [10, 63], is a 3-group paradigm that assigns different levels of uncontrollability to each group. Because the concept of control is central in learned-helplessness, the triadic design consists of 3 groups with differing levels of controllability: a master-escapable shock group, a yoked-inescapable shock group, and a control with no treatment group. There are 2 phases in the experiment; in the pretreatment phase when helplessness is experimentally induced, the master group experiences 30 unsignaled but escapable loud tones; the yoked group experiences 30 unsignaled inescapable loud tones, and the control group passively listens to loud tones identical to the ones used in the master and yoked groups. The triadic design allows us to manipulate the controllability of the identical stressor (loud tone, in our case), and because all three groups are exposed to the loud tones, any behavioral differences observed between groups would be due to the effects of coping rather than the stressor itself.

The computerized program of the task is written in PsychoPy and ran on Windows 10 system with a 23-inch monitor. The aversive stimulus is a 3000-Hz, 110db loud tone generated by PsychoPy, measured at 110db exact by an audiometer and played by two stereo speakers connected to the computer.

Upon arrival, participants are briefed that the whole study has three parts: one set of surveys and two computerized games, and it takes approximately one hour to finish. Participants are then asked to fill out one demographics survey, three anhedonia surveys (BDI-II, FCPS, & DARS), and one anxiety survey (STAI) as the baseline anhedonia and anxiety assessment. The participants to be recruited (n = 200) will be randomly assigned to 1 of the 3 treatment groups, matched in gender considering previous literature showing gender differences in learned helplessness behaviors [64–66]. In the pre-treatment phase, the master group receives loud tones but can escape by identifying and pressing the right sequence on the screen, and hence learn that the outcome is dependent on their responses and they gain a sense of controllability (controllability +1); in the yoked group, participants’ button presses have no effect on the aversive stimulus, meaning that the loud tone will be played for the full duration of each trial regardless of participant responses, so the yoked group will learn that the outcome is independent of their response, and hence they gain a sense of uncontrollability (controllability -1). Finally, the 3^rd^ control group is instructed to passively sit through and listen to all the tones and refrain from responding to the environment, so their overall sense of controllability remains unchanged over the situation (controllability = 0, or unchanged). In other words, control is not experimentally altered in the 3^rd^ control group as action-outcome learning is absent in this group. Participants are instructed that they will first play a game that has some unpleasant but not otherwise harmful loud noises. Participants are seated in front of the computer, and the master and the yoked groups are given the following verbal instructions:

“In the following task, from time to time a loud tone will come on for a while. When that tone comes on there is something you can do to stop it.”

The control group is given a different instruction:

“In the following task, from time to time a loud tone will come on for a while. Please sit and listen to it.

**Fig 1:**
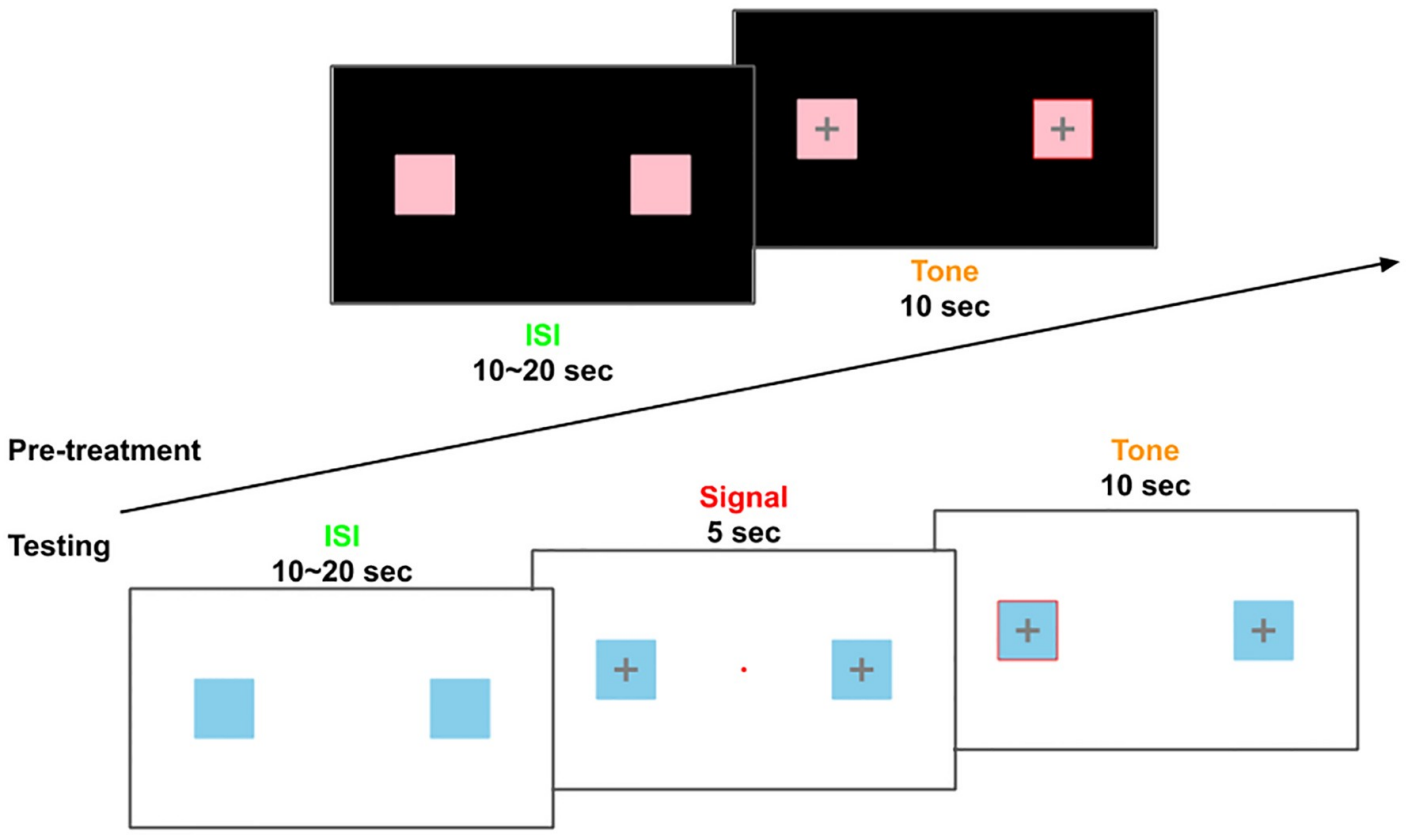
Schematic diagram of the LH task. Top: the pre-treatment phase; button, the test phase. **Pre-treatment**: after the ISI (could be any length between 10~20 seconds, with an average of 15 seconds), a loud tone appears simultaneously with a cross in each square and participants can press the squares to make a response. The correct response to terminate the tone is pressing the left square and then the right square/or right square then left square, alternating sides between trials (e.g. if the correct sequence is left-right in any given trial, the correct sequence in the next trial would be right-left). **Testing**: the correct sequence is left-right-left or right-left-right, alternating across trials.

Subsequently, a 3-sec sample of the loud tone is presented to the participants. Participants are told that the same tone will be used throughout the task, and they will immediately start with the **pretreatment session** after the sample tone is given. The **pretreatment session** consists of 30 unsignaled 10-sec trials during which the 3000-Hz, 110db tone is played. Throughout the **pretreatment session**, 2 colored squares are displayed on the screen with a black background (context A). Coinciding with the onset of the tone, a cross appears in each of the colored squares, signaling actions can be taken to stop the tone (Fig 1). The correct response to terminate the tone is pressing the left square and then the right square; in the next trial, the participant should reverse the order of the square by pressing the right square and then the left square. The correct sequences alternate across trials so if the participant correctly presses one sequence in any given trial, they should change to the other sequence in the next trial. After a while, the sequence of button pressing should become predictable to participants as they learn. A successful escape response is defined as pressing the correct sequence (left to right, or right to left depending on the given trial) and terminating the tone within 10 seconds. If participants fail to press the right buttons within 10 seconds, the tone and the buttons will disappear from the screen after the 10-sec interval and the trial is marked as failed. The inter-stimulus-interval (ISI) for the pretreatment session ranges 10–20 sec with an average ISI of 15-sec. Total numbers of success/fail trials as well as the response latency (defined in the next section) for all trials, are recorded. After completing 30 trials in the pretreatment session, participants take a 1-min break.

To test for helplessness, a **test session** is conducted after the 1-min break. Participants are again seated in front of the same computer, and all 3 groups are given the same instruction for the test session:

“In the following task, from time to time a loud tone will come on for a while. When that tone comes on there is something you can do to stop it.”

The **test session** is conducted using a similar, but not identical, computer task. In the test phase, the background color of the task changes from black to white, and the color of the squares also changes. These alterations signal a new context (context B), but the auditory stimulus is identical to the one used in the pretreatment session. The **test session** consists of 18 signaled 10-sec trials, with ISI ranges 10–20 sec with a mean of 15-sec. During each trial, a 5-sec red light and the four buttons on the screen appear at the center of the screen signaling the onset of the 10-sec tone, with the offset of the red light followed immediately by the onset of the loud tone. The appropriate escape response in the **test session is** different from that in the **pretreatment session**: participants need to press the left-right-left/right-left-right to terminate the tone, with the correct sequence alternating between trials. The participants are not explicitly told that the response sequence is different in the test session and need to learn to find the correct response as they proceed. Response latency is coded as follows: if the participants respond correctly between the onset and offset of the red light (latency of 5 sec or less), thus terminating the red light and the tone altogether, a successful *avoidance* trial is recorded; if the participants respond correctly after the offset of the red light but before the offset of the tone (latency of 5 to 15 sec), thus do not terminate the red light but terminate the tone within the 15-sec interval, a successful *escape* trial is recorded. If participants fail to respond correctly before the tone ends (latency larger than 15 sec), a *failed* trial is recorded.

Five behavioral measures are taken, including: (*a*) trials to the criterion for avoidance acquisition, defined as 3 consecutive avoidance responses; (*b*) trials to the criterion for escape acquisition, defined as 3 consecutive escapes (or a mix of 3 escape and avoidance trials); (*c*) the number of avoidance responses for the 18 trials (in the test phase); (*d*) the number of failures to escape, defined as the number of trials with a latency of 15 sec.; and (*e*) the overall mean response latency (defined as the time from the start of a trial until they make a correct response) for the 18 trials. If participants perform 2 successful escape responses and 1 successful avoidance response in succession, or 2 successful avoidance responses and 1 successful escape response, it is considered that they have met the criterion for escape acquisition but not the criterion for avoidance acquisition. The criterion for avoidance acquisition is only met when the participants have 3 consecutive successful avoidance responses.

At the conclusion of the test session, participants rate the level of aversiveness of the tone and take the *state* anhedonia surveys (FCPS and DARS) and *state* anxiety survey (STAI) again. We expect only participants from the yoked group to show increased *state* anhedonia and anxiety scores.

### The Signal-Detection Task (SDT)

To assess experimentally if participants’ hedonic capacity changes after acquiring learned helplessness, we will use the signal-detection reward task devised by Pizzagalli et al. [53], which is designed to assess reward responsiveness using laboratory-based measures. The rationale behind this task is that an unequal ratio of reward between two correct responses produced a response bias towards the more rewarded stimulus, rendering a preference to identify the more frequently rewarded stimulus, and reduced response bias to the more frequently rewarded stimulus is associated with a higher anhedonia score.

**Fig 2:**
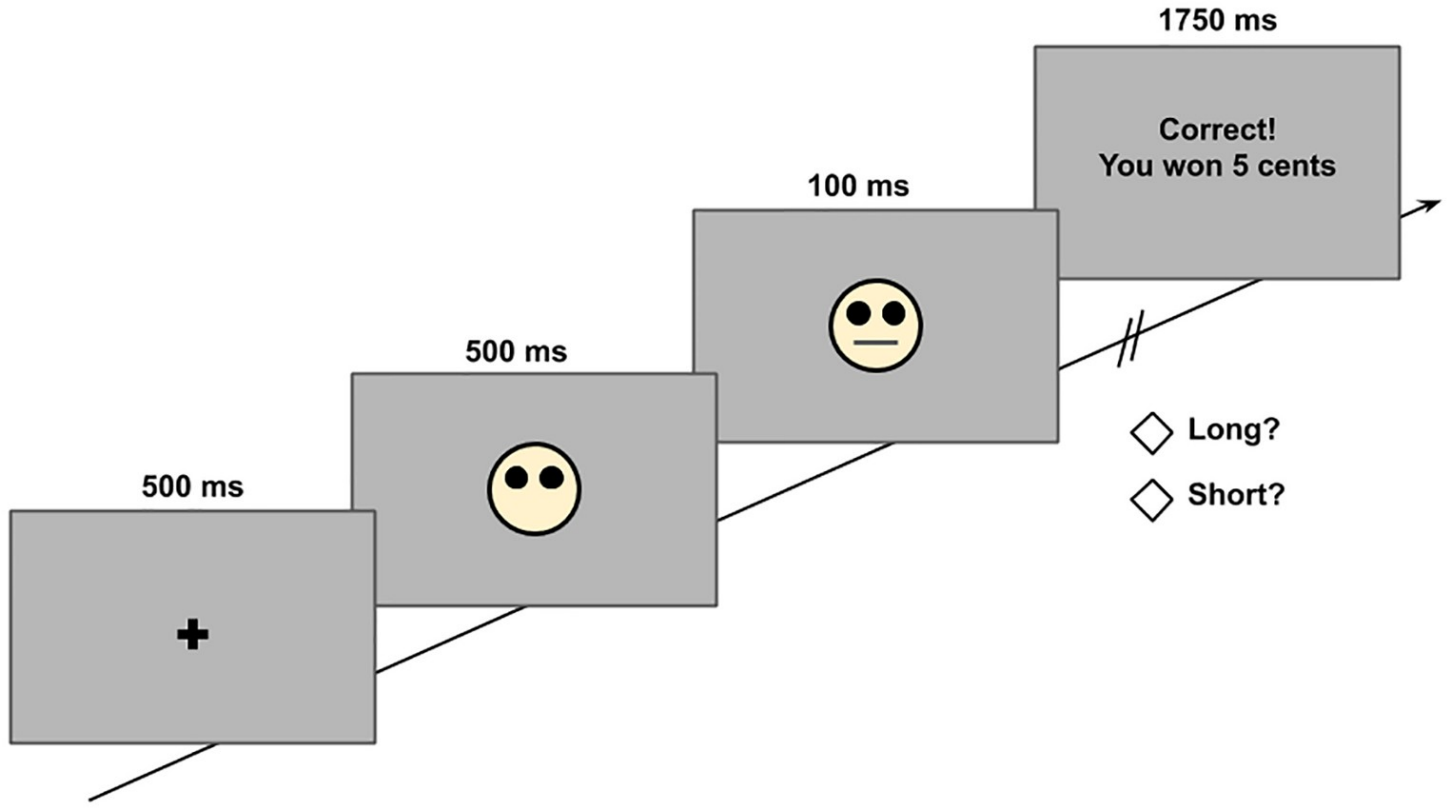
Schematic diagram of the SDT task. After a brief presentation of mouth in each trial, participants choose which type of stimulus they see by pressing either the “L” or the “S” key.

The task is written in computer software PsychoPy running on a Windows 10 computer. The task consists of four 50-trial blocks and lasts approximately 20 min. During each trial, an asterisk fixation point is shown at the center of the blank screen for 500 msec, followed by the presentation of a mouthless cartoon face in the center of the screen for another 500 msec. Immediately after, a short mouth (11.5 mm) or a long mouth (13 mm) is shown on the face for 100 msec only. Participants are then asked which mouth was shown and instructed to press either the “S” key or the “L” key to enter their response (counterbalanced across participants); the mouthless face stays on the screen until the participants’ response is entered. Although there is no time limit for each trial, participants are instructed to make their responses as fast as possible. If reward feedback is provided at a given trial, the text “Correct! You earned 5 cents!” is shown on the screen for 1750 msec after the correct response (Fig 2). The goal of the task is to choose the right face and earn as many points as possible, as the total number of points earned in the signal-detection task transfers to the number of raffle tickets participants can earn. All the raffle tickets will be drawn at the end of the chance to win a 100-dollar Amazon gift card. Participants are then given the written instructions and two sample trials before they proceed with the actual task.

The signal-detection task is based on a differential reinforcement ratio, meaning that an unequal number of positive feedback is given to one correct response vs. another correct response, and participants are told that not all correct responses will receive feedback. Specifically, only 20 out of 50 trials are followed by reward feedback in each block. For half of the participants (half from each of the 3 groups from the LH task, randomly assigned), correctly identifying the short mouth gives them three times more positive feedback (15 of 20, making the short mouth the “rich” stimulus) compared to correctly identifying the long mouth (5 of 20, making the long mouth the “lean” stimulus). The reward feedback contingency for the “rich” and “lean” stimulus is reversed for the other half of the participants. Asymmetrical reinforcement ratio is shown to be crucial to produce response biases (Johnstone & Alsop, 2000; McCarthy & Davison, 1979), and only positive feedback is given at any given trial to produce a positive response bias; no negative feedback is given if the wrong key is pressed at any given trial. The reward feedback is given following a pseudorandom schedule, and if the participants fail to correctly respond to a certain trial during which the reward feedback is scheduled, the reward feedback is delayed until the next correct identification is made.

Upon completing all the 200 trials, participants are debriefed, thanked, and given the course credits and corresponding numbers of raffle tickets for a chance to win a 100-dollar Amazon gift card.

We predict that the participants in the yoked group will have a blunted response bias towards the “rich” stimulus, demonstrating that learned helplessness will induce *state* anhedonia. We expect that the participants from the master and the control group will show a comparable response bias towards the “rich” stimulus, and there should be no significant differences in the response biases between the two groups.

### Ethical approval plan

Our study protocol has been approved by the University of Minnesota Institutional Review Board (IRB). The approval number is STUDY00005811. We will follow the IRB guidelines during and throughout the experiment. We will obtain participants’ written informed consent prior to the experiment and will store any identifiable data in a safe space in our facility. De-identified data will be made available and stored at the data repository of our University.

## Statistical analyses plan

### Main effects and interactions for the LH task

To examine if the yoked group has acquired learned helplessness after being exposed to unpredictable and uncontrollable loud tones, we will compare the mean response latency, trials to meet the criterion for avoidance/escape acquisition, the total number of successful avoidance trials, and the total number of failed trials between the 3 treatment groups. The response latency is grouped into 6 blocks of 3 trials each. A repeated-measure analysis of variance (ANOVA) test is used to test for the main effect and interactions of response latency across 6 blocks, with the treatment group as the between-subject measure and blocks as the within-subject measure. A post hoc Scheffe test will be performed to account for multiple comparisons if significance is reported. We predict that, compared to the master group and control group, the yoked group will have significantly longer response latency across 6 blocks.

A fixed-factor ANOVA is used to test for the main effects of (*a*) trials to the criterion for avoidance acquisition, defined as 3 consecutive avoidance responses; (*b*) trials to the criterion for escape acquisition, defined as 3 consecutive escapes; (*c*) the number of avoidance responses for the 18 trials; (*d*) the number of failures to escape, defined as the number of trials with a latency of 10 sec.; and (*e*) the overall mean response latency for the 18 trials, with treatment group as a between-subject measure. We predict that compared to the master group and control group, the yoked group will have *(1)* significantly more trials before they meet the avoidance acquisition criterion (i.e. slower learning speed), *(2)* significantly more trials before they meet the escape acquisition criterion, *(3)* significantly fewer numbers of successful avoidance trials, *(4)* significantly more failure trials, and *(5)* significantly longer average response latency.

### Main effects and interactions for the Signal-Detection Task (SDT)

Data will be analyzed with respect to the accuracy, discriminability, reaction time, and response bias. Accuracy is defined as the percent correct across 200 total trials. Reaction time (RT), defined as the time from the disappearance of the mouth until they make a successful key press, is the average RT calculated across 200 trials. Discriminability and response bias are both derived from the signal-detection behavioral model ([67, 68]; the detailed derivation can be found in the original paper [53] and the calculations are as follow:

Discriminability:

$$\text{log}\;d=\frac{1}{2}\;\text{log}\;(\frac{Long_{correct}*Short_{correct}}{Long_{incorrect}*Short_{incorrect}})$$

Response bias:

$$\text{log}\;d=\frac{1}{2}\;\text{log}\;(\frac{Long_{correct}*Short_{incorrect}}{Long_{incorrect}*Short_{correct}})$$

Where *Long*_*correct*_ is the number of correct responses after the presentation of the long mouth, *Short*_*incorrect*_ is the number of incorrect responses after the presentation of the short mouth, etc.

To test for accuracy and RT, we will use a repeated-measure ANOVA with stimulus Condition (lean or rich) and Blocks as repeated measure factors. For discriminability and response bias, only Block is included as the repeated measure. An analysis of covariance (ANCOVA) will also be performed to control for age and gender effects. To examine if acquired helplessness has an effect on response bias, we will also use the treatment group (yoked, master, and control) from the learned helplessness task as an additional between-subjects factor in the ANOVA. In addition, an analysis of covariance (ANCOVA) will be performed to control for age and gender effects. Post hoc Newman-Keuls tests will be performed in case of significant findings.

We predict that there will be no difference between the short and long mouth on average in all the three treatment groups, as the short and long mouth stimuli are counterbalanced and randomly assigned to participants as the “rich” or “lean” stimulus in all three groups. We expect to see a within-between subject interaction in response bias for Blocks and treatment group, such that the response bias for the “rich” stimulus should increase across blocks for the master and control groups, but not for the yoked group. In other words, we predict that the response bias will remain the same for the yoked group across blocks. We will also calculate the difference scores of response bias (Δ response bias) across blocks, or even across every 20 trials (e.g. Block 2 –Block 1, Block 3 –Block 2; trial 20~40 –trials 1~20, trial 40~60 –trial 20~40). We predict that the yoked group will show the smaller to no Δ response bias, while the master and the control group will show increased Δ response bias. We predict that the discriminability will remain unchanged for all 3 groups across blocks, and that all groups will find the task equally difficult.

### Main effects and interactions for the s*tate* anhedonic/anxiety surveys

To test if the hedonic and anxiety *state* change after the LH task, we will perform a one-way repeated measure ANOVA to compare the mean differences of the *state* anhedonic/anxiety scores for the 3 treatment groups. Post hoc Tukey test will be used if significance is reported.

We predict that both the anhedonia and anxiety *state* scores will increase after the LH task for the yoked group but not the master or the controlled group.

### Correlation between LH and the SDT task

Besides assessing how the factor treatment group affects the response bias, we are also interested in seeing if the mean response latency in the LH task correlates with response bias in the SDT task, collapsing data across the 3 treatment groups. If normality, linearity, and homoscedasticity assumptions are met, we will use a Pearson r correlation test to examine the correlation between average response latency (defined as the time from the start of a trial until they make a correct response) in the LH task and the response bias in the signal-detection task. By combining all data from all of the treatment groups and not dichotomizing the population (acquired LH or not), we will be able to extract more information from the data.

We predict that the average response latency in the LH task will be negatively correlated with response bias in the signal-detection task, such that people who have a longer average response latency in the LH task will more likely have a lower response bias score.

### Correlation between anhedonia surveys and the SDT task

To assess if the s*tate* anhedonic scores agree well with the results from the signal-detection task, we will perform a Spearman r correlation test to test each pair of the anhedonic score and the response bias score combination for all the 3 treatment groups if normality, linearity, and homoscedasticity assumptions are met.

We expect to see the s*tate* anhedonic scores agreeing with the anhedonic measure in the signal detection task, such that individuals with lower hedonic *state* scores (after the LH task) will have a lower response bias score, showing a positive correlation in the Spearman r correlation test.

### *Trait* depression/anxiety scores as covariates

To control for individual differences and how depression/anxiety *trait* scores impact how readily one acquires learned helplessness, we will do an Analysis of Covariance (ANCOVA) to control for *trait* scores and make sure any differences we see are not due to *trait* differences.

### Secondary analysis

Although we predict that the yoked group will develop learned helplessness and show performance deficits in all of the measures discussed above, we acknowledge the possibility that we may see alternative outcomes. Besides, there may exist certain confounding artifacts that may otherwise be responsible for the effects we see in our results. To account for all possible outcomes, we have implemented the following secondary analysis to better understand the main effects we get, or lack of effects if our predicted results are not observed. Individual differences in personality traits may affect who quickly and readily develops learned helplessness; if a person is highly anhedonic or anxious prior to the LH task, we predict that they will have a higher chance to show helplessness faster. To see how anhedonia and anxiety *traits* affect how easily one acquires learned helplessness, we will analyze the yoked group separately and see if the speed of developing learned helplessness is affected by their anhedonia and anxiety traits. We will perform 5 Spearman r correlation tests to examine the relationship between individuals’ anhedonia /anxiety *trait* scores and the following 5 measures: (*a*) trials to the criterion for avoidance acquisition, defined as 3 consecutive avoidance responses; (*b*) trials to the criterion for escape acquisition, defined as 3 consecutive escapes; (*c*) the number of avoidance responses for the 18 trials; (*d*) the number of failures to escape, defined as the number of trials with a latency of 10 sec.; and (*e*) the overall mean response latency for the 18 trials.

We expect that anhedonia and anxiety traits scores will be *(1)* positively correlated with the number of trials needed for avoidance acquisition; *(2)* positively correlated with the number of trials needed for escape acquisition; *(3)* negatively correlated with the total number of successful avoidance trials, *(4)* positively correlated with the number of failures to escape, and *(5)* positively correlated with the average response latency.

We think that anhedonia and anxiety traits scores could have an impact on any effects we see in the LH task, and it is possible that individuals with higher anhedonia and anxiety traits scores would have more severe performance impairments. However, with random assignment between groups, these pre-existing individual differences in personality traits should be balanced out. It is possible that tone perception can be a variable across individuals and that different individuals likely will have different tolerance to the loud tone, thus we have included a brief survey at the end of the LH task, asking the participants on a scale of 0–10, how annoying the participants would rate the tone. We will then perform an ANCOVA analysis to control for these individual differences in tone annoyance rating.

It is possible that different participants would rate the tone at different annoyance levels, which could impact their perception on how uncontrollable the situation is. But with random assignments at the beginning of the LH task, any pre-existing individual differences should be averaged out among groups. Thus, we predict that there will be no significant differences across participants on their tone annoyance rating and that their tone annoyance rating does not affect all the other behavioral results. As discussed in our introduction, we believe that attributional styles may play a role in the formation of helplessness. This is especially important if observed results do not follow our predictions (e.g. the yoked group does not show performance deficits in the LH task, or the performance deficits in the LH task do not transfer to the SDT task or the anhedonia surveys). If our predicted results are not observed, we think context specificity and attributional styles may account for the lack of effects in any one or more of our measures. To assess how attributional style contributes to the development of learned-helplessness, we will analyze participants’ ratings in the Attributional Style Questionnaire (ASQ). Specifically, we will look at how global and specific attributional styles correlate with the formation and transferability of helplessness. The reformulated LH theory (Abramson et al., 1978) states that participants learn to be helpless if they perceive the response-outcome non-contingency, but only if they make a correct attribution as the cause of their failure. In line with the reformulated LH theory, we believe that participants who had global attributional styles will show helplessness in both similar and new contexts; on the other hand, participants who had a specific attributional style may only show helplessness in a similar context, but this helplessness will not transfer to a new context. More specifically, we predict that participants who have specific attributional style will transfer the learned helplessness to the SDT task and the on pleasure scales, whereas participants who have a global attributional style may not show this transferable helplessness in the SDT task or/and on pleasure scales. We anticipate that the yoked participants will likely explore different kinds of strategies given their lack of control, and their behaviors will be more complex. Thus, and that response latency may not be enough to capture their exploratory behaviors. We are interested in understanding what kinds of strategies the yoked group uses, so we have the following measures to better assess the yoked participant’s behaviors. Specifically, we will perform exploratory analysis to examine the following data in both the conditioning and testing phases: 1. The number of different sequences the yoked group would try in any given trial, with a larger number of sequences tried indicating more strategies used; 2. The number of keypresses in each trial, with the more keypresses implying more active engagement and more attempts to escape; 3. On average, how many trials do the yoked participants try before they give up, defined by the number of trials before we see a steady decline in keypresses/or inactivity. The faster a participant is to give up faster in the conditioning phase suggesting they may be quicker to develop learned helplessness. These additional measures, together with response latency, should help us better assess participants’ behaviors during the LH task.

We add these additional measures to the conditioning as well as the testing phase because we think the conditioning phase may yield interesting results as this is when the yoked subjects would try the most strategies as they slowly start to realize their lack of control.

## Reliability, validity, & potential limitations

The current study aims to address the lack of research in human learned helplessness and its relationship with anhedonia. To experimentally induce learned helplessness in human participants, we designed a computer game using a triadic design, based on the original human version of the LH task [48, 62]. The goal of this task is to replicate the helplessness that was seen in the human original experiment, and we have a battery of anhedonia surveys as well as a signal detection task to assess participants’ anhedonia after the LH task. Although we design the new game as close to the original human LH task as possible, we realize that this experiment is not without weaknesses. We will name a few in this section.

Firstly, like most human helplessness experiments, this study has rather weak construct validity in its design (most LH tasks have a good face and predictive validity nonetheless). Unlike animal LH tasks, most human LH tasks, including this current protocol, do not confine participants in a truly uncontrollable environment for a prolonged period of time, nor do they use any traumatizing stimuli. Participants could terminate the experiment session anytime they wish, and this fact alone strongly attenuates the perception of uncontrollability. In addition, despite the performance deficits participants may demonstrate in the LH task, how can we be sure that participants truly develop helplessness in a lab setting over a short amount of time? Furthermore, it is hard to assess participants’ helplessness outside the lab setting, and how much the induced helplessness would transfer to real-life situations remains questionable. Thus, we would refrain from overgeneralizing the effectiveness of the current protocol; rather, any main effects we see in this study should be interpreted in lab settings and should not be viewed as permanent behavioral and neurochemical changes in the brain. For this reason, we focus on participants’ short-term behavioral changes and changes in state anhedonia and reward learning. Future studies should examine any long-term changes following such protocol to further determine the effectiveness of the current protocol.

A second limitation regards the general reliability of human LH tasks as some studies fail to produce performance impairments, or the performance deficits fail to transfer across situations [12, 20]. The reformulated LH theory [12] explains that the lack of effects in these studies should be interpreted by individual differences in attributional styles, such that participants only learn to be helpless if they make a correct attribution as the cause of their failure. If our predicted results are not observed, we think context specificity and attributional styles could account for the lack of effects in any one or more of our measures. To this end, we have implemented secondary analyses to examine participants’ attributional styles which improve the reliability of the current study.

Finally, there exists a divergence between non-human animal and human LH research, not only due to ethical considerations for human studies but also because the reformulated LH model is less testable in non-human animals because it is harder to examine cognition in non-human subjects [21, 22]. To bridge human and non-human LH research, future studies could devise new measures that capture the cognitive aspects of the reformulated LH mode.

## Timeline

The experiment (both the LH and the SDT task) is completed and coded using PsychoPy 3, and the questionnaires will be administered using the online survey platform Qualtrics. We have both the necessary support funding and facilities, as well as the approval (an approved IRB protocol) for the proposed research. Should the initial submission be accepted, we wish to start the data collection process right away, but due to the negative impact of the Covid19 pandemic and the fact that the University of Minnesota (our major participant pool) will only operate partially in-person in the foreseeable future, we expect that the data collection process will be much slower than we predicted. Originally, we estimated that it would take 6 months to collect all the data and another 3–4 months to perform data analysis, but given the current situation, we expect that the data collection process will take about a year or even longer depending on how soon the university campus reopens; once we have all the data, then we will need 4–6 months to process the data and complete the manuscript. In summary, we expect to have 16–18 months for the overall completion of the study, although this may change (either direction) depending on the evolution of the pandemic.

## Pilot data

We have some pilot data at the moment that was gathered in order to check how difficult and time-consuming the tasks are. This data will not be used for the analyses reported in the paper. Our goal is to start the data collection process as soon as we have approval from the PLOS ONE editorial board and our University permits.

## Data availability plan

De-identified data will be put online in a public repository. Specifically, we will upload the de-identified data to DRUM (Data repository for the University of Minnesota). The experiment and analysis code will be uploaded and made available at the Open Science Framework repository.
